# Supplementary material for: WordCommentsAnalyzer: A windows software tool for qualitative research
Source: F1000Res. 2018 Sep 4;7:536. Originally published 2018 May 3. [Version 2] doi: 10.12688/f1000research.14819.2 (PMC6137411; doi:10.12688/f1000research.14819.2)
Supplement: Supplementary file 3 [file f1000research-7-17614-s0002.tgz › b2a79496-4ada-47c4-9682-a99ce867836d.zip!/Eight Iranian Health Journals/Salmand_Iranian Journal of Ageing _4.docx]

© Salmand_Iranian Journal of Ageing . This work is licensed under CC BY-NC 4.0 (https://creativecommons.org/licenses/by-nc/4.0/).

Volume & Issue: دوره 12 - شماره 2

Title: سبک زندگی سالمندان مبتلا به استئوپروز و عوامل مرتبط با آن

http://salmandj.uswr.ac.ir/article-1-1252-fa.html

اهداف با توجه به روند پیرشدن جمعیت کشور و نقش بسیار مهم سبک زندگی در بیماری‌های مزمن و شایع سنین سالمندی به‌خصوص استئوپروز، مطالعه حاضر با هدف بررسی سبک زندگی سالمندان مبتلا به استئوپروز طراحی و اجرا شد. مواد و روش ها این مطالعه، مقطعی و از نوع توصیفی‌تحلیلی است که در سال ۱۳۹۲ روی ۳۰۰ نفر از سالمندان مبتلا به استئوپروز مراجعه‌کننده به مراکز سنجش تراکم استخوان دانشگاه علوم پزشکی تهران انجام شد. نمونه‌گیری به روش در دسترس انجام شد و اطلاعات با استفاده از پرسش‌نامه سنجش سبک زندگی سالم در سالمندان ایرانی جمع‌آوری شد. تجزیه‌وتحلیل داده‌ها با استفاده از نرم‌افزار SPSS نسخه ۲۱ انجام شد. برای توصیف نمونه‌ها از آمار توصیفی، آزمون تی‌تست و تجزیه‌وتحلیل واریانس استفاده شد (P≤۰/۰۵). یافته ها این مطالعه روی ۳۰۰ نفر از سالمندان مرد (۳۷/۹ درصد) و زن (۶۲/۱ درصد) با میانگین سنی ۵/۶۳±۶۷ انجام شد. نتایج بیانگر آن است که بیشتر سالمندان مبتلا به استئوپروز (۷۱/۳ درصد) سبک زندگی متوسط، ۱۰/۷ درصد سبک زندگی مطلوب و ۱۸ درصد سبک زندگی نامطلوب دارند. یافته‌های پژوهش نشان داد بین میانگین کلی نمره سبک زندگی با سن (۰/۴۹۹=P) و جنس (۰/۱۷۶=P) در سالمندان مبتلا به استئوپروز از نظر آماری تفاوت معنی‌دار وجود ندارد، ولی بین میانگین کلی نمره سبک زندگی با وضعیت تأهل (۰/۰۰۱=P)، سطح تحصیلات (۰/۰۲۷=P) و ابتلا به بیماری مزمن (۰/۰۰۹=P) از نظر آماری تفاوت معنی‌دار وجود دارد. نتیجه گیری با توجه به اینکه سبک زندگی درصد اندکی از سالمندان مبتلا به استئوپروز در سطح مطلوب قرار دارد، این مسئله قابل توجه و تأمل است و نیازمند بررسی‌های بیشتر و برنامه‌ریزی مسئولان در سطح کلان برای برطرف‌شدن مشکلات مذکور در سبک زندگی سالمندان مبتلا به استئوپروز است.

Title: مشکلات اقدامات درمانی از دیدگاه مراجعان سالمند مراکز بهداشتی‌درمانی شهر همدان (مطالعه‌ای کیفی)

http://salmandj.uswr.ac.ir/article-1-1113-fa.html

اهداف افزایش سن با ابتلا به بیماری‌ها ارتباط دارد و باعث افزایش مشکلات درمانی در سالمندان می‌شود. با توجه به افزایش سن جمعیت و نیاز به مصرف دارو، شناخت چالش‌های سلامت و مشکلات اقدامات درمانی در سالمندان مهم است. این مطالعه کیفی برای شناسایی مشکلات اقدامات درمانی از دیدگاه سالمندان در شهر همدان انجام شده است. مواد و روش ها مطالعه کیفی حاضر با رویکرد تحلیل محتوا انجام گرفت. داده‌ها از طریق مصاحبه عمیق نیمه‌ساختاریافته با ۱۵ سالمند مراجعه‌کننده به مراکز بهداشتی شهر همدان در سال ۱۳۹۴ جمع‌آوری شد. روش نمونه‌گیری مبتنی بر هدف بود و از هر دو جنس با تنوع سنی، وضعیت اقتصادی‌اجتماعی و میزان تحصیلات، اطلاعات جمع‌آوری شد. برای تجزیه‌و‌تحلیل از روش تحلیل محتوا استفاده شد. شرکت‌کنندگان و محققان استحکام داده‌ها را تأیید کردند. یافته ها سالمندان مراجعه‌کننده با میانگین سنی ۶۶/۴۷ و انحراف معیار ۴/۴۷ سال بودند. از ۱۵ سالمند شرکت‌کننده ۶ نفر زن و ۹ نفر مرد بودند. تحلیل داده‌ها منجر به استخراج سه درون‌مایه اصلی به عنوان چالش‌های مصرف دارو در سالمندان شد که شامل علل مربوط به پزشکان، مشکلات مصرف دارو و کمبود داروها و یازده درون‌مایه فرعی هزینه معاینه، بی‌اعتمادی به پزشکان، اهمیت‌ندادن پزشکان به سالمندان، عوارض داروها، زود تمام‌شدن داروها، اثربخشی داروها، نبود داروهای جایگزین، متعددبودن داروها، استفاده از داروهای دیگران، نبود داروها در برخی از داروخانه‌ها و تحویل داروهای تاریخ‌گذشته به سالمندان بود. نتیجه گیری نتایج مطالعه نشان داد در سالمندان مشکلات اقدامات درمانی وجود دارد. بنابراین می‌توان پس از بررسی مشکلات، با آموزش و مداخلات مناسب، به سالمندان در این زمینه راهنمایی و کمک کرد. مسئولان نیز می‌توانند از یافته‌های این پژوهش برای برنامه‌ریزی استفاده کنند.

Title: سال‌خوردگی جمعیت در ایران و هزینه‌های رو به افزایش بهداشت و درمان

http://salmandj.uswr.ac.ir/article-1-1226-fa.html

اهداف هدف از انجام این مطالعه، تحلیل الگوی مصرف هزینه‌های بهداشت و درمان در چرخه زندگی خانوارهای شهری ایران با تأکید بر دوران سال‌خوردگی، طی سال‌های 1375 تا 1391 است. مواد و روش ها این مطالعه از نوع تحلیل ثانویه است که با استفاده از داده‌های بخش جمعیت سازمان ملل متحد و داده‌های هزینه درآمد خانوار و به کمک روش شبه‌تابلویی انجام پذیرفت. به منظور بازآفرینی و تجزیه‌وتحلیل داده‌ها از نرم افزار STATA استفاده شد. همچنین برای تعدیل مقدار هزینه‌ها نسبت به بُعد خانوار و سن افراد خانوار، از شاخص مقیاس معادل و به منظور تورم‌زدایی از سطح هزینه‌ها در سال‌های مختلف، از شاخص قیمت مصرف‌کننده استفاده شد. یافته ها یافته‌های پژوهش نشان داد جمعیت ایران در حال گذار از دوران جوانی به سال‌خوردگی است، به‌نحوی‌که تعداد سالمندان در سال 1414 به بیش از 10 میلیون نفر و درصد سال‌خوردگی به بیش از 11 درصد خواهد رسید. تحلیل اثر سن بر هزینه‌های بهداشت و درمان حاکی از آن است که تقاضای مصرف از سن 25 تا 50 سالگی به شکل نسبتاً ملایمی در حال افزایش است. پس از آن شیب خیلی تندی به خود می‌گیرد و در سال‌های پایانی عمر به اوج خود می‌رسد. اثر نسل نشان داد نسل‌های جدید در مقایسه با نسل‌های قبل، در زمینه هزینه‌های بهداشت و درمان تقاضای مصرف بیشتری دارند. همچنین نتایج حاصل از بررسی اثر سال نشان‌دهنده این است که تقاضای مصرف طی سال‌های بررسی‌شده، در ابتدا ثابت و سپس حالت افزایشی و کاهشی داشته است.  نتیجه گیری بر اساس نتایج این پژوهش می‌توان گفت افراد در طول چرخه زندگی همواره درصدی از هزینه کل خود را به هزینه‌های بهداشتی و درمانی اختصاص می‌دهند، اما درصد این تخصیص در سنین مختلف متفاوت است؛ به‌نحوی‌که با افزایش سن و به‌ویژه در دوران سالمندی، تقاضا در بخش هزینه‌های بهداشت و درمان افزایش قابل توجهی خواهد یافت. همچنین در سطح کلان، با توجه به افزایش درصد سال‌خوردگان در جمعیت ایران طی دهه‌های آینده، سهم هزینه‌های بهداشت و درمان از هزینه کل افزایش خواهد یافت.

Title: رابطه نیازهای اساسی روان‌شناختی با سرزندگی ذهنی در سالمندان

http://salmandj.uswr.ac.ir/article-1-1149-fa.html

اهداف سرزندگی ذهنی داشتن وضعیت مثبت، سرشار از انرژی و بانشاط بودن است. چنین حالتی در دوره سالمندی اهمیت فراوانی دارد. بنابراین، هدف پژوهش حاضر بررسی رابطه نیازهای اساسی روان‌شناختی با سرزندگی ذهنی در سالمندان بود. مواد و روش ها پژوهش حاضر کاربردی و توصیفی از نوع مقطعی است. جامعه آماری این تحقیق تمامی سالمندان زن و مرد شهر تهران بودند که در مجموع ۲۳۵ شرکت‌کننده شامل ۱۰۰ زن و ۱۳۵ مرد به طور در دسترس انتخاب شدند و با مقیاس‌های سرزندگی ذهنی ریان و فردریک و نیازهای اساسی روان‌شناختی دسی و ریان آزموده شدند. برای تجزیه‌وتحلیل داده‌ها در بخش آمار توصیفی از شاخص‌های میانگین، انحراف استاندارد و در بخش آمار استنباطی از تحلیل رگرسیون چندگانه گام‌به‌گام استفاده شد. برای تجزیه‌وتحلیل داده‌ها از نرم‌افزار آماری SPSS۲۳ استفاده شد. یافته ها نتایج تحلیل همبستگی و رگرسیون گام‌به‌گام به ترتیب نشان داد بین متغیرهای خودفرمانی و سرزندگی ذهنی ارتباط معنادار و مثبتی وجود دارد (P<۰/۰۱، r=۰/۶۰). بین متغیرهای شایستگی و سرزندگی ذهنی نیز ارتباط مثبت و معناداری وجود دارد (P<۰/۰۱، r=۰/۵۳). همچنین، بین متغیرهای ارتباط و سرزندگی ذهنی ارتباط معنادار و مثبتی وجود دارد (P<۰/۰۱، r=۰/۵۸). متغیرهای ﺧﻮدفرمانی، ارتباط و شایستگی می‌توانند ۳۱ درصد از واریانس سرزندگی ذهنی در سالمندان را تبیین و پیش‌بینی کنند.   نتیجه گیری برآورده‌شدن نیازهای اساسی روان‌شناختی در سالمندان به سرزندگی ذهنی آن‌ها منجر می‌شود. بنابراین شناخت نیازهای اساسی روان‌شناختی و توجه به آن‌ها می‌تواند راهکاری مؤثر برای حفظ سرزندگی ذهنی سالمندان باشد.

Title: تأثیر تمرین‌های آمادگی جسمانی و تمرین ذهنی و تمرین ذهن‌آگاهی بر تعادل ایستا و پویای زنان سالمند

http://salmandj.uswr.ac.ir/article-1-1259-fa.html

اهداف سالمندان در معرض اختلالاتی در حفظ تعادل هستند که منجر به افتادن و آسیب‌دیدگی آن‌ها می‌شود. مطالعه حاضر تأثیر تمرین‌های آمادگی جسمانی، تمرین ذهنی و تمرین ذهن‌آگاهی را بر بهبود تعادل ایستا و پویا در زنان سالمند بررسی کرده است. مواد و روش ها شرکت‌کنندگان شامل 60 زن 60 تا 75 سال بودند که به صورت تصادفی در چهار گروه (سه گروه تجربی تمرین ذهنی، ذهن‌آگاهی، آمادگی جسمانی و یک گروه کنترل) (هر گروه 15 نفر) قرار گرفتند. برای اندازه‌گیری تعادل پویا و ایستا از آزمون‌های تعادل بِرگ و شارپند رومبرگ استفاده شد. گروه‌های تجربی به مدت هشت هفته (هفته‌ای سه جلسه 75 دقیقه‌ای) در تمرین‌های آمادگی جسمانی و تمرین ذهنی و ذهن‌آگاهی شرکت کردند. سپس پس‌آزمون و پس از گذشت هشت هفته، آزمون پیگیری گرفته شد. برای تجزیه‌وتحلیل داده ها از آزمون تحلیل واریانس برای اندازه‌های مکرر و تحلیل واریانس یک‌طرفه در سطح معنی‌داری 0/05 استفاده شد. یافته ها در پس‌آزمون نمره‌های تعادل (ایستا و پویا) بین گروه‌های تمرین ذهن‌آگاهی و تمرین ذهنی اختلاف معنی‌داری وجود نداشت (P>0/05)، ولی تأثیر تمرین‌ها در گروه تمرین ذهن‌آگاهی و گروه تمرین ذهنی در مقایسه با گروه‌های تمرین آمادگی جسمانی و کنترل، مثبت و معنی‌دار بود (P<0/001). همچنین تأثیر تمرین‌های گروه آمادگی جسمانی نیز نسبت به گروه کنترل مثبت و معنی‌دار بود (P<0/001). در آزمون پیگیری نمره‌های تعادل (ایستا و پویا) بین گروه‌های تمرین ذهن‌آگاهی و تمرین ذهنی اختلاف معنی‌داری وجود نداشت (P>0/05). همچنین بین گروه‌های تمرین آمادگی جسمانی و کنترل نیز اختلاف معنی‌داری وجود نداشت (P>0/05). تأثیر تمرین‌ها در گروه تمرین ذهن‌آگاهی و گروه تمرین ذهنی در مقایسه با گروه‌های تمرین آمادگی جسمانی و کنترل، مثبت و معنی‌دار بود (P<0/001). نتیجه گیری نتایج نشان داد تمرین‌های آمادگی جسمانی، تمرین ذهن‌آگاهی و تمرین ذهنی در بهبود تعادل (ایستا و پویا) زنان سالمند مؤثر است. با این حال همراهی تمرین‌های شناختی (تمرین ذهنی و تمرین ذهن‌آگاهی) با تمرین‌های حرکتی (تمرین‌های آمادگی جسمانی) موجب ایجاد تأثیر ماندگار می‌شود. بنابراین توصیه می‌شود از تمرین‌های آمادگی جسمانی به همراه تمرین‌های شناختی در بهبود تعادل (ایستا و پویا) سالمندان استفاده شود.

Title: بررسی وضعیت افسردگی، حمایت اجتماعی ادراک‌شده و برخی عوامل مرتبط با آن‌ها در سالمندان ساکن شهر تهران در سال 1393

http://salmandj.uswr.ac.ir/article-1-1157-fa.html

اهداف افزایش جمعیت سالمندان پیامد بهبود بهداشت عمومی است و توجه به سلامت جسمی و روانی آنان موضوعی اجتناب‌ناپذیر است. هدف این مطالعه تعیین وضعیت افسردگی، حمایت اجتماعی ادراک‌شده و برخی عوامل مرتبط با آن‌ها در سالمندان ساکن شهر تهران در دانشگاه علوم پزشکی شهید بهشتی است. مواد و روش ها این مطالعه مقطعی با ۵۸۰ سالمند ساکن شهر تهران در حوزه دانشگاه علوم پزشکی شهیدبهشتی و نمونه‌گیری چند‌مرحله‌ای نظام‌مند با گرفتن رضایت آگاهانه از شرکت‌کنندگان انجام شد. بیماری نورولوژیک، ابتلا به اختلالات روانی شناخته شد و سابقه بستری به علت بیماری‌های روانی معیار خروج از مطالعه بوده است. اطلاعات باپرسش‌نامه‌های استاندارد افسردگی بک وحمایت اجتماعی ادراک‌شده زیمت و پرسش‌نامه اطلاعات جمعیت‌شناختی جمع‌آوری و با نرم افزار Stata و روش‌های آماری توصیفی و تحلیلی (من ویتنی و کروسکالوالیس و آزمون همبستگی اسپیرمن) تحلیل شد.  یافته ها میانگین سن شرکت‌کنندگان ۷/۹۱±۶۹/۶۶ سال محاسبه شد. ۶۷/۵۳ درصد از آنان فقط با همسرانشان زندگی می‌کردند. ۴/۰۱ درصد بی‌سواد و ۸۹/۶۹ درصد حداقل صاحب یک منزل مسکونی بودند. پوشش بیمه درمان ۹۲/۳۸ درصد محاسبه شد. ۲۹/۶ درصد از نظر اقتصادی و ۴۶/۹۹ درصد از افراد نیز از زندگی خود ابراز رضایت کردند. شیوع افسردگی خفیف، متوسط و شدید به ترتیب ۱۷/۴۱، ۸۸/۵۸، ۸/۲۵ با میانگین ۵۱/۸۷ درصد و موافقت با حمایت اجتماعی ادراک‌شده نیز ۷۲/۳۸ درصد به دست آمد. آزمون کروسکال والیس نشان داد سطوح مختلف افسردگی با حمایت اجتماعی ادراک‌شده (۰/۰۰۱>P) رابطه معنی‌داری دارد. آزمون همبستگی نیز نشان داد با افزایش حمایت اجتماعی ادراک‌شده میزان افسردگی کاهش یافته است (۰/۳۸۸-). متغیرهای وضعیت تأهل، مسکن، بیمه درمان، رضایت از زندگی و رضایت اقتصادی با هر دو متغیر افسردگی و حمایت اجتماعی رابطه معنی‌داری نشان داد. تحصیلات با افسردگی رابطه معنی‌دار آماری داشت، ولی بین جنسیت با افسردگی و نیز تحصیلات و جنسیت با حمایت اجتماعی ادراک‌شده ارتباط معنی‌داری برقرار نبود. نتیجه گیری فراوانی افسردگی و موافقت با حمایت اجتماعی ادراک‌شده در سالمندان زیاد است. عوامل اقتصادی و خانوادگی مشترکی با افسردگی و حمایت اجتماعی مرتبط است. تلاش برای بهبود این عوامل مشترک، بهبود وضعیت افسردگی و حمایت اجتماعی درک‌شده را به همراه خواهد داشت و سالمندی سالم‌تری را به ارمغان می‌آورد.

Title: تحلیل محتوایی و استنادی مقالات چاپ‌شده در مجله علمی‌پژوهشی سالمندی ایران 1385 تا 1394

http://salmandj.uswr.ac.ir/article-1-1316-fa.html

اهداف از آنجا که نشریات علمی‌پژوهشی یکی از کارآمدترین ابزارهای اطلاع‌رسانی در تبادل اطلاعات علمی در جهان هستند، لازم است به منظور شناسایی و کنترل فعالیت‌های پژوهشی، این نوع نشریات به صورت دوره‌ای ارزیﺎﺑﯽ شوند. مطالعه حاضر به منظور تحلیل محتوایی و استنادی مقالات چاپ‌شده در مجله علمی‌پژوهشی سالمندی ایران در سال‌های ۱۳۸۵ تا ۱۳۹۴ انجام شد. مواد و روش ها مطالعه حاضر با روش توصیفی به بررسی و تحلیل محتوایی و استنادی تمام مقالات چاپ‌شده در مجله سالمندی ایران طی سال‌های ۱۳۸۵ تا ۱۳۹۴ پرداخته است. برای جمع‌آوری داده‌ها از چک‌لیست محقق‌ساخته تحلیل محتوایی و استنادی مقالات چاپ‌شده در مجله علمی‌پژوهشی سالمندی ایران استفاده شد. به منظور تجزیه‌وتحلیل داده‌ها از روش‌های آمار توصیفی و تحلیلی با کمک نرم‌افزار SPSS نسخه ۲۳ استفاده شد. یافته ها از ۲۹۸ مقاله چاپ‌شده، حیطه ورزش و فعالیت بدنی با ۱۸/۵ درصد و پس از آن مسائل روانی و جسمی با ۱۲/۴ و ۱۰/۴ درصد به ترتیب بیشترین سهم از مقالات چاپ‌شده را داشتند. بیشترین روش استفاده‌شده در مطالعات، روش توصیفی‌تحلیلی با ۶۱ درصد و کمترین روش استفاده‌شده در مطالعات، روش کارآزمایی بالینی با حدود ۱/۷ درصد بود. در خصوص پراکندگی مقالات، حدود ۵۷ درصد از مقالات از تهران بود. درصورتی‌که از ۵ استان خراسان شمالی، کردستان، کهگیلویه‌وبویراحمد، هرمزگان و همدان هیچ مقاله‌ای چاپ نشده بود. میانگین مدت‌زمان دریافت تا چاپ مقاله حدود ۲۸۴ روز با انحراف‌معیار ۱۴۳ روز بود. حدود ۲۲ درصد از مقالات با میانگین ۲/۲۶ و انحراف‌معیار ۱/۹۵ مرتبه استناد شده بودند. آزمون‌های تحلیلی نشان داد بین عواملی چون دوره‌های مختلف و محل انجام پژوهش با میزان استنادات رابطه معنادار وجود دارد. نتیجه گیری با توجه به نتایج حاصل از مطالعه حاضر، برای ارتقای کمّی و کیفی مجله علمی‌پژوهشی سالمند، پیشنهاد می‌شود دست‌اندرکاران مجله به منظور توزیع متعادل حیطه‌های پژوهشی مبتنی بر اهداف مجله، کاهش مدت‌زمان دریافت تا چاپ مقاله و توزیع مناسب پراکندگی جغرافیایی مقالات اقداماتی انجام دهند.

Title: اثربخشی معنادرمانی گروهی بر اضطراب مرگ و امید به زندگی سالمندان مقیم مراکز شبانه‌روزی شهر کرمان

http://salmandj.uswr.ac.ir/article-1-1184-fa.html

اهداف با توجه به روند روبه‌رشد جمعیت سالمندان، رفع مشکلات آن‌ها ازجمله مشکلات روانی، ضروری به نظر می‌رسد. از آنجا که افزایش اضطراب مرگ و کاهش امید به زندگی در دوران سالمندی شایع‌تر است، پژوهش حاضر با هدف بررسی اثربخشی معنادرمانی گروهی بر اضطراب مرگ و امید به زندگی سالمندان مقیم مراکز شبانه‌روزی شهر کرمان انجام شد. مواد و روش ها جامعه آماری این پژوهش شامل تمامی سالمندان مقیم مراکز شبانه‌روزی شهر کرمان بود. از این جامعه دو مرکز امیرالمؤمنین و مهرآذین به ‌طور تصادفی انتخاب شد و سپس با مراجعه به این دو مرکز، 30 نفر (18 زن و 12 مرد) به طور تصادفی انتخاب و به دو گروه آزمایش و کنترل (هر کدام 15 نفر) تقسیم شدند. به گروه آزمایش، معنادرمانی گروهی در ده جلسه (هر جلسه 90 دقیقه) آموزش داده شد. گروه کنترل در این مدت مداخله‌ای دریافت نکردند. هر دو گروه قبل و بعد از اجرای آزمایش به پرسش‌نامه امید به زندگی میلر (1997) و مقیاس اضطراب مرگ ((BDI پاسخ دادند. داده‌ها با استفاده از روش‌های آماری نظیر فراوانی، درصد، میانگین، انحراف استاندارد و تحلیل کواریانس چندمتغیره تجزیه‌و‌تحلیل شدند. یافته ها نتایج نشان داد معنادرمانی گروهی می‌تواند بر اضطراب مرگ سالمندان مقیم مراکز شبانه‌روزی در سطح معناداری 01/P<0 تأثیر داشته باشد. همچنین معنادرمانی گروهی می‌تواند بر امید به زندگی سالمندان مقیم مراکز شبانه‌روزی در سطح معناداری 01/P<0 تأثیر داشته باشد. نتیجه گیری بر اساس نتایج این پژوهش، معنادرمانی گروهی می‌تواند برای کاهش اضطراب مرگ و افزایش امید به زندگی سالمندان مقیم مراکز شبانه‌روزی و افزایش سلامت روانی آنان استفاده شود. با توجه به تأکیدهای خاص معنادرمانی بر زمان حال و همچنین معنای رنج برای سالمندان، استفاده از آن در ارتقای سلامت اجتماعی سالمندان اهمیت خاصی دارد.

Title: بررسی ارتباط راهبردهای مقابله و منبع کنترل با اضطراب مرگ در سالمندان

http://salmandj.uswr.ac.ir/article-1-1276-fa.html

اهداف اضطراب مرگ در افراد سالمند شایع است. هدف از پژوهش حاضر بررسی ارتباط راهبردهای مقابله و منبع کنترل با اضطراب مرگ در سالمندان است. مواد و روش ها در این مطالعه توصیفی‌مقطعی، ۱۱۳ سالمند بالای ۶۰ سال در شهر تهران به روش نمونه‌گیری در دسترس انتخاب شدند. برای گردآوری داده‌ها مقیاس راهبردهای مقابله‌ای، مقیاس چندبعدی منبع کنترل و مقیاس اضطراب مرگ در گروه نمونه پژوهش اجرا شد. سپس داده‌ها با نسخه ۲۱ نرم‌افزار SPSS و آزمون‌های آماری ضریب همبستگی پیرسون و رگرسیون گام‌به‌گام تجزیه‌وتحلیل شد. یافته ها میانگین سنی شرکت‌کنندگان ۳/۹۳±۶۵/۱۹ بود. میانگین و انحراف استاندارد مقابله مسئله‌مدار، مقابله هیجان‌مدار، مقابله اجتنابی، منبع کنترل درونی، منبع کنترل بیرونی و اضطراب مرگ به ترتیب ۷/۴۵±۴۸/۲۰، ۶/۹۲±۵۰/۹۳، ۸/۶۷±۵۰/۳۹، ۵/۷۰±۱۷/۸۵، ۶/۰۸±۶۱/۵۱ و ۲/۳۱±۸/۶۷ بود. بر اساس یافته‌های پژوهش بین راهبردهای مقابله‌ای و منبع کنترل با اضطراب مرگ در سالمندان رابطه معنی‌داری وجود دارد (۰/۰۵>P). نتایج تحلیل رگرسیون نشان داد کنترل بیرونی، مقابله اجتنابی و مقابله هیجان‌مدار به طور معنی‌داری اضطراب مرگ در سالمندان را پیش‌بینی می‌کنند (۰/۰۱>P). نتیجه گیری اضطراب مرگ یکی از عوامل مؤثر در سلامت روان سالمندان است. با توجه به یافته‌ها، راهبردهای مقابله و منبع کنترل نقش مهمی در اضطراب مرگ سالمندان دارند. بنابراین، با طراحی مداخلات روان‌شناختی بر اساس راهبردهای مقابله و تغییر منبع کنترل از بیرونی به درونی، سلامت اضطراب مرگ را می‌توان بهبود بخشید.

Title: بررسی میزان شیوع سوء رفتار با سالمندان در ایران از سال 1384 تا 1394 با استفاده از مرور ساختاریافته و متاآنالیز

http://salmandj.uswr.ac.ir/article-1-1308-fa.html

اهداف یکی از آسیب‌های اجتماعی که امروزه جوامع با آن روبه‌رو هستند، پدیده سالمندآزاری است که می‌تواند عوارضی نظیر کاهش اعتمادبه‌نفس، احساس ناامیدی، بی‌تفاوتی و مشکلات روحی روانی و ناتوانی را برای سالمند در پی داشته باشد. با انجام این مطالعه و ادغام مطالعات مختلف که در ایران انجام شده است، می‌توان به برآیندی کلی دست یافت و به وسعت مشکل که همان شیوع کلی سالمندآزاری است، پی برد. مواد و روش ها این مطالعه با روش مرور نظام‌مند و متاآنالیز انجام شده است که در آن 55 مقاله که درباره سالمندآزاری در ایران از سال 1384 تا 1394در پایگاه‌های ایران‌مدکس، مگیران، گوگل‌اسکالر، اس‌آی‌دی، و پابمد وجود داشت، جمع‌آوری شد. درنهایت 10 مقاله که واجد شرایط تجزیه‌وتحلیل بودند در نرم‌افزار Comprehensive Meta-Analysis (V2.0, Biostat) تجزیه‌وتحلیل شدند. یافته ها بیشترین شیوع مقطعی کلی سوء رفتار با سالمندان در مطالعه منوچهری و همکاران (87/8 درصد) در تهران و کمترین آن در مطالعه حسینی و همکاران (17/1 درصد) در سال 1394 در شهرکرد بود. برآیند میزان کلی شیوع سالمندآزاری در ایران در این مطالعه 56/4 درصد با فاصله اطمینان 95 درصد (5/75-1/35) با استفاده از مدل تصادفی است. نتیجه گیری میزان شیوع کلی سالمندآزاری در ایران 56/4 درصد بود که این میزان در مقایسه با مطالعاتی که در خارج انجام شده، بسیار بیشتر است. این ناهمخوانی با وجود تعریف یکسان از سالمندآزاری و استفاده از پرسش‌نامه تقریباً یکسان و روش همانند را می‌توان بیشتر به علت حجم نمونه‌های کم، داشتن خرده‌فرهنگ‌ها ونگرش‌های مختلف اقوام دانست. میزان شیوع سالمندآزاری در ایران بیشتر از کشورهای دیگر است.
